# Maternal and perinatal outcomes after labor induction in Robson Group 5 patients

**DOI:** 10.61622/rbgo/2026rbgo75

**Published:** 2026-10-30

**Authors:** Bárbara Scalco Geller, Roxana Knobel, Thais Carloto Todeschini, Alberto Trapani, Karine Souza da Correggio

**Affiliations:** 1 Universidade Federal de Santa Catarina Florianópolis SC Brazil Universidade Federal de Santa Catarina, Florianópolis, SC, Brazil.

**Keywords:** Induced labor, Cesarean section, Vaginal birth after cesarean, Uterine rupture, Pregnancy outcome

## Abstract

**Objective:**

To analyze the characteristics and mode of delivery of pregnant women in Robson group 5 attended at a public hospital in Santa Catarina who underwent labor induction, investigating predictive factors for vaginal delivery and maternal-infant outcomes.

**Methods:**

Retrospective observational descriptive study conducted at the Maternity Ward of the University Hospital of the Federal University of Santa Catarina between January 2020 and December 2024. A total of 323 Robson group 5 pregnant women who underwent labor induction were included. Variables analyzed included mode of delivery, skin color, previous vaginal delivery, indication for induction and cesarean section, cervical dilation on admission, comorbidities, use of Foley catheter, complications, Apgar score, and neonatal ICU admission. Chi-square test, Student's t-test, and logistic regression were performed.

**Results:**

The induction success rate was 37.2%. The main indication for cesarean section was failed induction (36%), followed by cephalopelvic disproportion (25.6%) and non-reassuring fetal status (23.6%). Previous vaginal delivery and Foley catheter use showed statistical significance for vaginal delivery outcome (p<0.001 and p=0.004, respectively). Newborns delivered by cesarean section had significantly higher mean birth weight (3570.1 g vs. 3359.1 g, p<0.001). In logistic regression, absence of previous vaginal delivery increased the odds of cesarean section nearly threefold (OR=2.97; 95%CI: 1.70–5.17), and non-use of Foley catheter doubled this probability (OR=2.22; 95%CI: 1.28–3.86). Ten uterine ruptures occurred (3.1%), with no maternal or fetal mortality.

**Conclusion:**

Labor induction in Robson Group 5 patients was associated with acceptable maternal and neonatal outcomes, although success rates were lower than those reported in international literature. Institutional protocols incorporating Foley catheter use and prenatal counseling strategies are recommended to promote informed decision-making regarding mode of delivery.

## Introduction

The marked increase in cesarean section rates in Brazil has direct repercussions on maternal and infant health and on public policies for obstetric care. Cesarean rates exceeding 10% are not associated with reductions in maternal and neonatal mortality.^(1,2)^ Nevertheless, in 2023, nearly 60% of births in Brazil were by cesarean section,^(3)^ representing one of the highest rates worldwide. This reality is further underscored by the significant disparity between the public and private sectors, wherein women attended in the private sector present cesarean rates more than double those of women who deliver through the public health system, suggesting the influence of structural and cultural factors on the decision regarding mode of delivery.^(4)^

Although cesarean section is an essential procedure, its performance without medical indication is associated with short- and long-term maternal and neonatal risks, compromising women's reproductive health and increasing the likelihood of complications in future pregnancies. Prevention of the first cesarean section is fundamental to controlling the growth of cesarean rates, given that the primary indication for repeat cesarean is a prior cesarean delivery.^(5,6)^

Since 2014, the World Health Organization (WHO) has recommended monitoring cesarean section rates using the Robson classification, a system that categorizes pregnant women into ten groups based on obstetric criteria, enabling the evaluation of care practices and the development of strategies to reduce unnecessary cesarean sections.^(5,7)^ The groups with the greatest impact, representing 70% of cesarean sections performed in Brazil, are group 2 (nulliparous women admitted not in labor), group 10 (pregnancies under 37 weeks), and group 5 (multiparous women with a previous cesarean and a term pregnancy). Group 5 alone accounts for approximately 20% of the Brazilian obstetric population and is responsible for nearly one-third of all cesarean sections performed in the country.^(4,8)^

Trial of labor after cesarean (TOLAC) represents an important strategy to contain this trend. When properly indicated and safely conducted, vaginal birth after cesarean (VBAC) constitutes a safe alternative compared to elective repeat cesarean section. Evidence demonstrates that VBAC can be successful in up to 70% of cases, reducing maternal complications and favoring postpartum recovery. For this reason, promoting VBAC is fundamental to reducing the incidence of cesarean sections in Brazil. Despite this, TOLAC rates have declined substantially worldwide in recent years.^(9–11)^

In certain clinical situations, interruption of pregnancy prior to the spontaneous onset of labor becomes necessary due to maternal or fetal conditions that preclude the safe continuation of gestation. Labor induction represents an obstetric resource employed in such circumstances; however, its use in women with a prior cesarean section poses a particular challenge. The presence of a prior uterine scar requires careful evaluation of risks and benefits, considering both the safety of the procedure and its success rates, given that failed induction may result in a repeat cesarean section and its associated risks.^(9,12)^

In this context, the present study aims to analyze the characteristics and mode of delivery of pregnant women in Robson group 5 who attended a public hospital in Santa Catarina and underwent labor induction, investigating predictive factors for vaginal delivery and maternal-infant outcomes.

## Methods

This was a retrospective observational descriptive study conducted at the Maternity Ward of the Polydoro Ernani de São Thiago University Hospital of the Federal University of Santa Catarina (HU-UFSC/EBSERH), using data from January 2020 to December 2024. Pregnant women in Robson group 5 who underwent labor induction were included. Exclusion criteria comprised records with more than 50% missing data, admissions for abortion or uterine curettage, and records with data inconsistencies. Data were extracted from the institutional maternity database, populated from the labor and birth data recording form completed during obstetric hospitalization. All information obtained was anonymized.

The categorical variables included were mode of delivery, self-reported skin color, previous vaginal delivery, indication for induction, indication for cesarean section, cervical dilation on admission, presence of chronic hypertension, diabetes mellitus, premature rupture of membranes, post-term pregnancy, use of catheter for induction, and presence of maternal and fetal complications. Failed induction was not collected as an independent variable but as one of the categories within the indication for cesarean section variable, as registered in the medical record; adherence to the institutional induction protocol could not be verified from the available secondary data. Continuous variables were neonatal birth weight, maternal age, gestational age, and cervical dilation on admission.

Data were analyzed using IBM SPSS Statistics software, version 27. Descriptive statistical analysis was initially performed, with absolute and relative frequencies for categorical variables and measures of central tendency and dispersion for continuous variables. For comparisons between groups (vaginal delivery versus cesarean section), the chi-square test was used for categorical variables and Student's t-test for continuous variables, after verifying homogeneity of variances with Levene's test. Subsequently, logistic regression was performed using the enter method, including variables that were statistically significant in the bivariate analysis, to assess the odds ratio for vaginal delivery. Missing data were flagged in the results and excluded from analyses. A significance level of 5% was adopted.

The study was approved by the Research Ethics Committee of HU-UFSC/EBSERH (CAAE: 90884125.8.0000.0121), in accordance with Resolution 466/2012 of the National Health Council. Waiver of the Informed Consent Form (ICF) was requested as provided for in CNS Resolution No. 510/2016, given the retrospective nature of the study and the exclusive use of anonymized secondary data. Potential selection bias was minimized by including all eligible cases during the study period.

## Results

During the study period, 7,330 births occurred at the institution, of which 1,316 (17.9%) were patients in Robson Classification Group 5. Of these, 341 (25.9%) were initially included because labor induction was their obstetric management. After applying eligibility criteria, 323 (24.5%) patients remained in the final analyzed sample. Eight patients were excluded for having more than 50% of their medical record information absent, and 10 patients were excluded for having no recorded delivery outcome (Figure 1).

**Figure 1:**
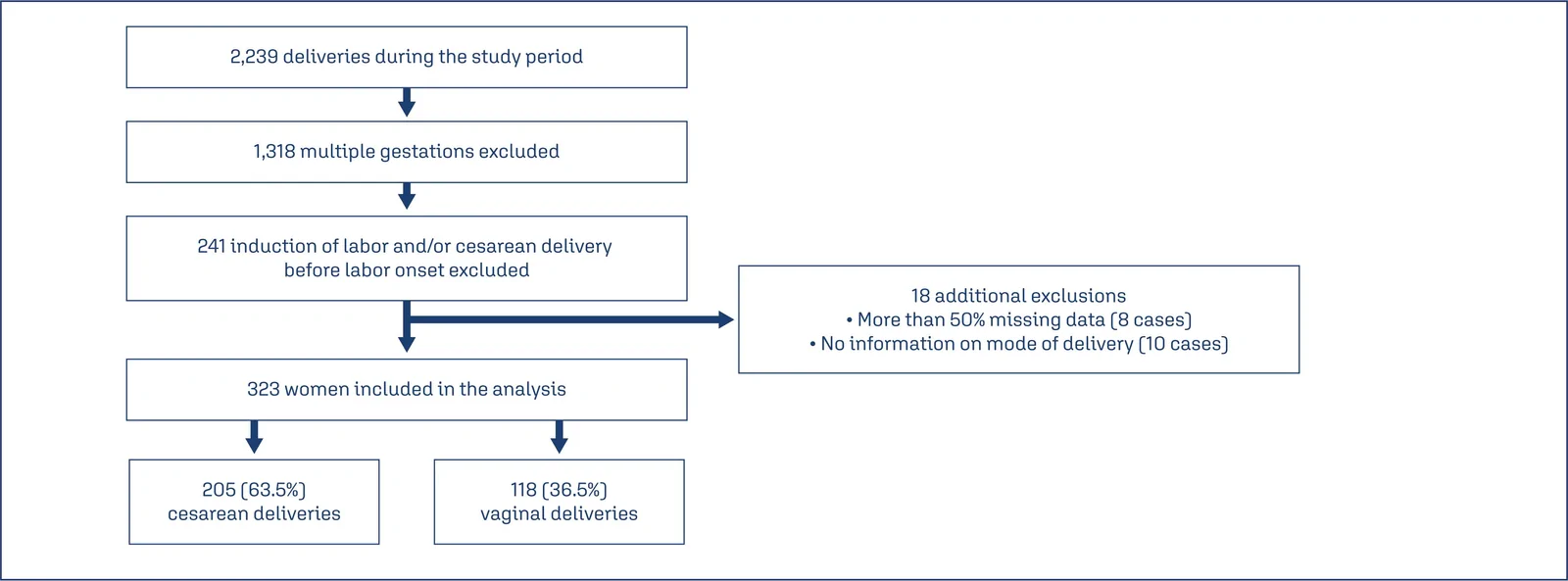
Study participant selection

Regarding the demographic and obstetric characteristics of the analyzed sample, the majority of patients were white (65.9%), had no previous vaginal delivery (79.9%), and had no prior history of miscarriage (76.2%). The primary indication for labor induction was post-term pregnancy, present in 37.5% of participants, followed by premature rupture of membranes (16.4%) and gestational diabetes mellitus (12.4%) (Table 1). Mean maternal age was 30.15 years (standard deviation [SD] = 5.96), while gestational age at admission had a mean of 39.76 weeks (SD = 1.31). Regarding cervical status at hospital admission, 48.3% of patients presented with a closed cervix, 36.5% had dilation between 1 and 2 cm, and only 7.1% had dilation greater than 2 cm.

**Table 1:** Population characteristics

Characteristics		Mean	SD
Maternal age (years)		30.15	5.96
Gestational age (weeks)		39.76	1.31
**Variables**			**n(%)**
Self-reported skin color*			
	White		213(65.9)
	Brown (Parda)		45(13.9)
	Black		34(10.5)
	Asian		5(1.5)
Previous vaginal delivery			
	0		258(79.9)
	1		40(12.4)
	>1		25(7.8)
Indication for induction			
	Post-term pregnancy (≥41 weeks)		121(37.5)
	Premature rupture of membranes		53(16.4)
	Gestational diabetes mellitus		40(12.4)
	Hypertensive disorders		37(11.5)
	Maternal diseases		14(4.3)
	Oligohydramnios		11(3.4)
	Other indications		23(7.1)
Cervical dilation on admission**			
	0 cm		156(48.3)
	1–2 cm		118(36.5)
	>2 cm		23(7.1)

SD: standard deviation.

* 26 patients with missing skin color data at admission.

** 26 patients with missing cervical dilation data at admission. &Other induction indications: fetal alteration (3); fetal growth restriction (5); poor obstetric history (6); other unspecified indications (9).

The success rate for vaginal birth after cesarean (VBAC) induction was 37.2%. The primary indication for cesarean section in Robson Group 5 patients was failed induction (36%), followed by cephalopelvic disproportion (25.6%) and non-reassuring fetal status (23.6%). When considering only the 250 participants who effectively entered active labor, the VBAC success rate following induction was 48%. Maternal request accounted for 8.9% of cesarean indications in these patients (Table 2).

**Table 2:** Indications for cesarean section in women with a prior cesarean section submitted to labor induction

Variables	n(%)
Failed induction	73(36.0)
Cephalopelvic disproportion / arrest of labor progress	52(25.6)
Non-reassuring fetal status	48(23.6)
Maternal request	18(8.9)
Other unspecified indications	9(4.4)
Abnormal fetal presentation	2(1.0)
Uterine rupture	1(0.5)

Regarding favorable vaginal delivery outcomes, the presence of one or more previous vaginal deliveries and the use of Foley catheter for labor induction showed statistical significance (p<0.001 and p=0.004, respectively). Maternal comorbidities such as hypertensive disorders and gestational diabetes, as well as premature rupture of membranes and post-term pregnancy, showed no association with mode of delivery outcome. Although 73.2% of cesarean sections performed were in Caucasian patients, skin color also showed no statistical significance (p=0.46) (Table 3).

**Table 3:** Sociodemographic characteristics, obstetric and clinical history, and use of catheter for induction according to mode of delivery

Variables	Vaginal delivery n(%)	Cesarean section n(%)	p-value
White (Caucasian) skin color	79(69.3)	134(73.2)	0.46
Previous vaginal delivery	42(35.0)	34(16.7)	<0.001*
Hypertensive disorders / preeclampsia	15(12.5)	32(15.8)	0.42
Diabetes mellitus / gestational	16(13.3)	28(13.8)	0.90
Premature rupture of membranes	21(18.8)	31(16.6)	0.63
Post-term pregnancy	45(37.5)	86(42.4)	0.40
Use of Foley catheter for induction	40(33.3)	39(19.2)	0.004*

* Statistically significant value.

In the comparison of continuous variables by mode of delivery (Table 4), a statistically significant difference was observed only for birth weight. Newborns delivered by cesarean section had a higher mean weight (3570.1 ± 482.5 g) compared to those delivered vaginally (3359.1 ± 424.1 g), with a mean difference of 211 g (p<0.001). The other variables assessed maternal age, gestational age, and cervical dilation on admission showed no significant differences between groups. The boxplot (Figure 2) illustrates these findings, showing a higher median and greater dispersion in birth weight among neonates born by cesarean section, including extreme values consistent with macrosomia.

**Table 4:** Comparison of continuous variables according to mode of delivery

Variable	Mode of delivery	n	Mean	SD	Mean diff.	p-value*
Birth weight (g)	Vaginal	116	3359.1	424.1	−211.0	<0.001
	Cesarean	200	3570.1	482.5		
Maternal age (years)	Vaginal	119	29.9	5.9	−0.51	0.461
	Cesarean	194	30.4	6.0		
Gestational age (days)	Vaginal	119	279.8	9.3	−0.98	0.347
	Cesarean	201	280.8	8.8		
Cervical dilation on admission (cm)	Vaginal	110	0.88	1.12	0.18	0.136
	Cesarean	185	0.70	0.91		

SD: standard deviation.

* Student's t-test for independent samples with assumed equal variances, except for cervical dilation on admission (Levene's test p=0.014).

**Figure 2:**
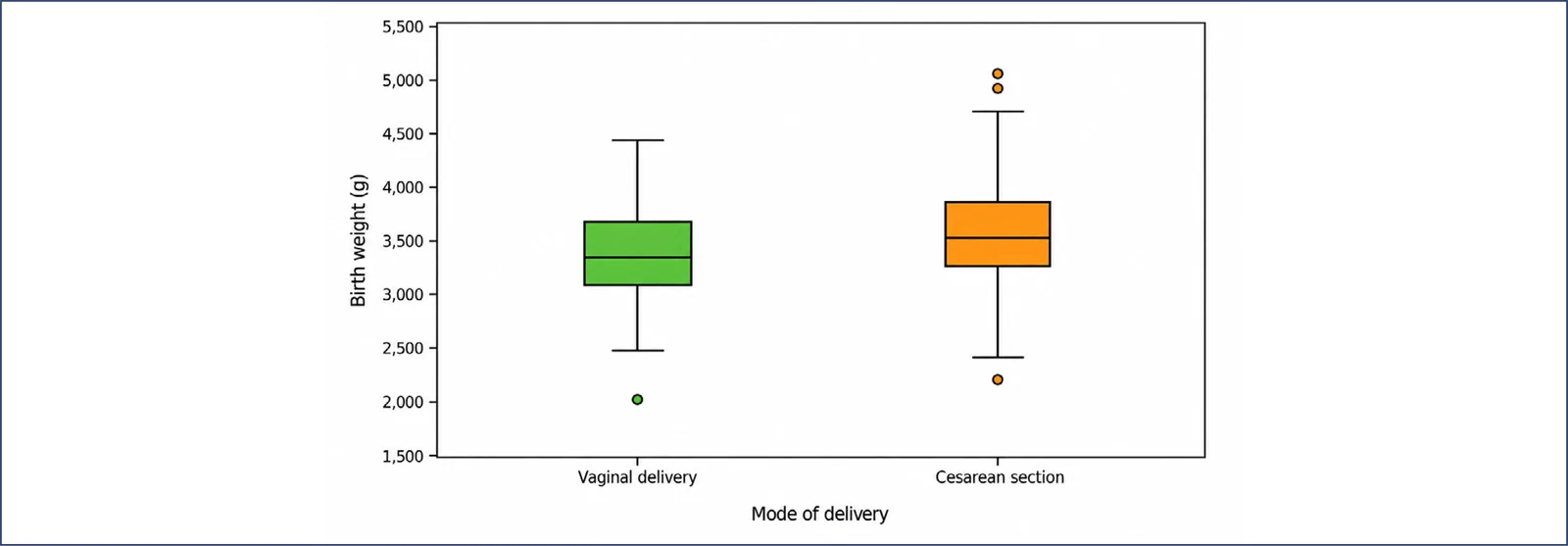
Distribution of neonatal birth weight according to mode of delivery

Complications occurred in 22 cases (6.9%). Two cases of shoulder dystocia were recorded, one involving a neonate weighing over 4,000 g. One case of postpartum hemorrhage following vaginal delivery and one case of bladder injury during cesarean section were also noted. Ten cases of uterine rupture occurred, one of which was diagnosed following a vaginal delivery. In only one case was cesarean section indicated due to the diagnosis of uterine rupture; the remaining cases were identified intraoperatively, with cesarean section indicated for failed induction (5 cases) and arrest of labor progress (3 cases). Among these ten cases, no neonate required neonatal ICU admission, and no maternal severe morbidity was documented. There were no fetal or maternal deaths. In total, eight neonates were admitted to the neonatal ICU: two born by vaginal delivery and six by cesarean section.

In the logistic regression analysis, the absence of a prior vaginal delivery was strongly associated with the cesarean section outcome, increasing the odds of this mode of delivery by nearly threefold (adjusted OR=2.97; 95%CI: 1.70–5.17). Similarly, non-use of catheter for induction was associated with a higher probability of cesarean section, with approximately twice the odds compared to patients who used this method (adjusted OR=2.22; 95%CI: 1.28–3.86). The model included the variables previous vaginal delivery, catheter use, and neonatal birth weight, showing adequate calibration by the Hosmer-Lemeshow test (p=0.51).

## Discussion

This study demonstrated that the profile of Robson Group 5 patients submitted to labor induction was consistent with the literature, characterized predominantly by women without a previous vaginal delivery. Post-term pregnancy was the primary indication for the procedure, reflecting current practice of avoiding continuation of pregnancy beyond 41 weeks, when perinatal risks progressively increase. The other indications observed, such as premature rupture of membranes, diabetes, and hypertension, are consistent with recommendations that contraindicate continuation of pregnancy in the presence of maternal or fetal risks.^(13–16)^

The VBAC success rate of 37.2% was lower than those reported in international studies, where rates ranged from 43.7% to more than 70%.^(14,16,17)^ A previous study conducted at the same institution between 2013 and 2016 found a success rate of 43.4%,^(18)^ a figure higher than that observed in the present study. Failed induction was the primary cause of cesarean section, a result that differs from Brazilian studies involving patients with a prior cesarean, which identify non-reassuring fetal status as the leading cause.^(19,20)^ This finding warrants a methodological note: the institutional protocol defines failed induction as the absence of active labor onset following maximum oxytocin dose, amniotomy, and at least 12 to 18 hours after membrane rupture. However, the retrospective nature of this study did not allow verification of protocol adherence on a case-by-case basis, since time elapsed between membrane rupture and surgical decision, oxytocin dose records, and amniotomy documentation were not systematically retrievable from secondary data. This limitation may introduce information bias in either direction: toward overestimation, if the indication was registered before full protocol criteria were met, or toward underestimation, if some cases were reclassified under other indications such as arrest of labor progress.

Maternal request accounted for 8.9% of cesarean indications in our sample. This finding warrants particular attention, considering that in Thailand, despite high VBAC induction success rates, only 28% of pregnant women accept attempting TOLAC,^(21)^ and that approximately 90% of maternal requests for cesarean section occur in patients with a prior cesarean.^(22)^ The main reasons cited for maternal choice of cesarean section include fear of pain during labor, perception of greater safety due to maternal and fetal risks, prior traumatic experiences, and knowledge deficits.^(23,24)^ These factors could be adequately addressed through appropriate counseling, promoting more informed decision-making regarding mode of delivery and ensuring adequate obstetric support for vaginal birth.^(25–27)^ In China, it was possible to reduce elevated elective cesarean rates through strategies such as health education, mandatory written informed consent, labor analgesia, continuous support during birth, and targeted training of obstetric nurses and obstetricians.^(28)^

History of previous vaginal delivery was identified as a predictor of VBAC success. The absence of prior vaginal delivery tripled the probability of subsequent cesarean section. These results converge with the international literature, which consistently demonstrates prior vaginal delivery as the primary factor associated with successful induction in patients with a previous cesarean section.^(11,19,21,22,29)^

Use of a Foley catheter was a factor that increased the odds of vaginal delivery in this sample. Evidence on the safest and most effective induction methods in pregnant women with a prior cesarean section is limited due to heterogeneity of outcomes and the small number of high-quality randomized controlled trials.^(30)^ Balloon catheter use as a mechanical induction method is considered safe and effective, with vaginal delivery rates between 50% and 61%.^(31,32)^ Due to concerns that prostaglandins may increase the risk of uterine rupture in patients with a prior uterine scar, this method is not used at our institution, although several authors have employed low doses with success and safety.^(17,33)^ The combined use of oxytocin with another method increases the probability of successful labor induction.^(16,34)^ A study comparing induction methods in Robson Group 5 patients showed that vaginal delivery rates with oral misoprostol and Foley catheter were similar (70%), with no significant difference in uterine rupture rates between methods.^(17)^

Neonates born by cesarean section had a significantly higher mean birth weight compared to those born vaginally, with a statistically significant difference of 211 g, suggesting that higher fetal weight may be a limiting factor for successful vaginal delivery following induction.^(14,16,22)^ However, fetal weight was no longer a significant variable in the adjusted logistic regression model. Information on the indication for the prior cesarean section was not available in the database; this variable could have influenced the observed outcomes, as demonstrated by several studies, particularly those related to dystocia, fetal macrosomia, and maternal characteristics.^(14,16,22)^

In the analyzed population, several variables showed no significant association with vaginal delivery success. Premature rupture of membranes demonstrated no association with the outcome, a result supported by a study that also found no statistical difference,^(17)^ although other studies report that this condition increases success rates for vaginal delivery.^(22)^ Maternal age showed no significant association with induction success, diverging from a meta-analysis that identified advanced maternal age as a factor associated with a higher probability of cesarean section.^(11)^ Similarly, patients with Black skin color differed from results reported in a US study demonstrating a higher risk of cesarean section in Black women undergoing induction, a finding attributed to lower rates of shared decision-making during labor.^(35,36)^

Cervical dilation at admission also did not prove to be a predictor of vaginal delivery, diverging from studies demonstrating that higher Bishop scores are associated with higher success rates.^(19,22,29)^ The inability to systematically record the complete Bishop score at hospital admission represents a limitation of the present study. Maternal comorbidities (hypertension/preeclampsia and diabetes mellitus/gestational) showed no significant relationship with vaginal delivery success. However, studies show an indirect association, linking younger patients with a lower prevalence of comorbidities and greater frequency of trial of labor to higher vaginal delivery success rates.^(11,19,20)^ This negative influence of maternal comorbidities may reflect the clustering of cardiometabolic risks, with the need for earlier labor induction and a lower probability of spontaneous labor, consistent with studies associating maternal age, body mass index, and metabolic disorders with the risk of cesarean section.^(19,21,37,38)^

The primary concern related to labor induction in Robson Group 5 patients is the risk of uterine rupture. This event occurred in 10 cases (3.1%) in the present study, with no associated maternal morbidity or mortality. This result is consistent with other articles on labor induction in pregnant women with a prior cesarean section, which demonstrated no difference in obstetric morbidity among women submitted to induction, even though these patients present a higher risk of uterine rupture compared to those with spontaneous labor.^(13,15)^

Regarding neonatal outcomes, the neonatal ICU admission rate in the present study was 2.5% (8/323), lower than the 4.7% reported in a Brazilian cohort of SUS newborns in São Paulo.^(39)^ We found no significant differences in Apgar scores or neonatal ICU admission rates between groups. This result diverges from studies demonstrating that induction was associated with a higher risk of adverse perinatal outcomes, with more frequent resuscitation in the delivery room among neonates born to mothers with induced labor, though without resulting in higher perinatal mortality.^(13,40)^ A Brazilian study published in 2013 identified, as perinatal outcomes, a higher frequency of transient tachypnea of the newborn, respiratory dysfunction, and prolonged neonatal ICU stay in patients undergoing cesarean section, while the VBAC group showed a significant association with Apgar scores below seven at five minutes and neonatal hypoxia, although neonatal mortality was similar between groups.^(20)^ These findings differ from those of the present study, which found no statistically significant differences in neonatal complications. The present study has limitations related to the use of secondary data, including the inability to fully retrieve certain information, such as the indication for the prior cesarean section, the possibility of missing data in other variables, and the lack of a complete Bishop score record at hospital admission. However, notable strengths include the substantial sample size (n=323) specific to Robson Group 5 — a group in which increasing cesarean indication rates have been observed in Brazil. The results demonstrate the feasibility and safety of labor induction for this patient group, with no maternal or fetal mortality and an acceptable complication rate.

## Conclusion

The findings demonstrate that labor induction in Robson Group 5 patients is a safe procedure, although with a lower success rate than reported in international studies. Previous vaginal delivery and use of Foley catheter were factors associated with VBAC success. Efforts to improve induction success rates should include developing institutional protocols for Foley catheter use, as well as education and counseling strategies for pregnant women during prenatal care to promote informed decision-making regarding the mode of delivery.

## Data Availability

The research data are described in the article presented.
